# The Foreign Journals

**Published:** 1836-07

**Authors:** 


### Art. XVIII.—THE FOREIGN JOURNALS. No. III.

### DANISH JOURNALS.

4. *Samlinger til den Danske Medicinal Historie, udgivne af* Dr. J. D. Herholdt og Dr. F. V. Mansa. *Kjobenhavn.* 8vo.

*Collections in the Medical History of Denmark, &c.*

This journal, which was edited by the veteran Professor Herholdt, recently deceased, and Dr. Mansa, is not on the plan of ordinary journals, although it is published in that form. It comes out irregularly, if it may not be termed an annual, since the first three numbers bear date 1833, 1834, 1835, respectively. It is entirely devoted to the illustration of the medical literary history of Denmark, and is therefore chiefly interesting to the profession in that country. It is a small work; the three first numbers only constituting a single octavo volume of 354 pages. Like most of the productions of our northern brothers, this work is of solid value, containing many historical notices and other interesting articles. We fear the death of its principal editor will be fatal to its continuance.

Besides the Medical Journals, strictly so called, published in the Danish language, (the whole of which we have noticed,) reviews of medical books are also occasionally to be found in two other periodical works chiefly devoted to general literature, the *Maanedskrift for Literatur*, and the *Dansk Literaturtidende;* the former published monthly, as its name implies, and conducted by twelve literary men; the latter published weekly, and without any avowed editor.

GERMAN JOURNALS.

9.—*Medicinische Zeitung. Herausgegeben von dem Verein für heilkunde in Preussen.—Berlin.* Fol. 1836.
*Medical Gazette, &c.*

This journal possesses somewhat of a more corporate character than the other German Journals, inasmuch as it was set on foot by the Medical Society termed "The Prussian Medical Association," and is still the official organ of that institution. It is under the superintendence of a committee of the Society, consisting at present of the following members: J. N. Rust, Eck, and Grossheim.

The exterior of this journal reminds one of the original edition of the old Spectator, which one now and then meets with in the libraries of collectors. It consists of a single folio sheet, about the size of a common sheet of foolscap writing-paper, and consequently contains but four pages: an awkward supplement, consisting of a single loose leaf, is however frequently given; not a little indicative of the old-fashioned and slovenly habits of "The Trade" in Germany. The Gazette appears weekly, and costs yearly three dollars and two-thirds of a dollar, (about ten shillings of our money.) It is now in its fifth year, having been begun in Sept. 1832. It is a plain, sober journal, and somewhat of the gravest for a weekly publication. Beside the usual matters contained in the larger journals, it justifies its title of *Gazette* by giving various little pieces of medical news interesting to the profession; such as appointment to offices, the conferring of honours, changes of residence, deaths, &c. A marked and interesting feature in the character of the *Zeitung* is the numerous extracts which it gives from the official reports of the civil medical officers in the pay of government, distributed throughout the Prussian dominions, and which are furnished to the *Association* by the authorities. On the whole, the work is authentic and sound, and consequently valuable.

10.—*Zeitschrift für die Ophthalmologie.* Herausgegeben von Dr. F. A. von Ammon, Professor an der Chirurgisch-medicinischen Akademie zu Dresden, &c.—*Heidelberg und Leipzig.* 8vo.
*Journal of Ophthalmology.* Edited by Dr. F. A. von Ammon, Professor in the Medico-chirurgical Academy at Dresden, &c. —*Heidelberg and Leipzig.*

The taste—we should say, rather, the enthusiasm,—which prevails among the medical practitioners of Germany for the study of the eye, has doubtlessly been powerfully promoted by the periodical publications of that country, which, more or less devoted to the subject, have been conducted by men of the most distinguished talents and experience. We may mention particularly the *Ophthalmologische Bibliothek,* commenced in 1802, by Himly and Schmidt; the *Neue Bibliothek für die Chirurgie und Ophthalmologie,* by Langenbeck, in 1818; the *Journal der Chirurgie und Augen-Heilkunde,* by Graefe and Walther, in 1820; and, though last not least, the work now before us. A mine of truths is contained in these works, in examining which the reader may rise, time after time, with the renewed feeling of wonder that so many interesting enquiries have been instituted regarding the structure, functions, and diseases of a single organ, the study of which is still far from being exhausted. Happy should we be, could we stimulate our British students to some small share of the spirit which has so long distinguished their brethren in Germany, and which is still stimulating to the elaboration of new and important facts the successors of Haller, and Zinn, and Wrisberg, and Meckel, and Soemmerring, and Beer, and Prochaska.

This journal has existed since the year 1830, and has reached its fifth volume. It is published quarterly, the four parts forming an annual volume, price three dollars. Each part contains from eight to nine sheets, or 120 to 140 pages; it is printed in the Roman type, and generally contains illustrative plates. It is composed of original memoirs, selections from other journals, and analytical and critical notices of works. It is pretty strictly confined to ophthalmology, and is conducted with great judgment by its learned editor, who has long been distinguished as one of the first ophthalmologists in Germany. It is a most valuable publication, and one which will supply us with much important matter.

### AMERICAN JOURNALS.

4.—*The American Journal of Pharmacy, published by authority of the Philadelphia College of Pharmacy.* Edited by R. E. Griffith, M.D.—*Philadelphia.* 8vo. 1836.

This journal had reached its twenty-fourth number, under the name of "The Journal of the Philadelphia College of Pharmacy," when this was changed, in April last, for the title above transcribed. It is published quarterly; each number consisting of five and a half sheets, and four Nos. forming an annual volume; the price, two dollars and a half. As the advertisement on its cover states, it "is devoted to the objects of pharmaceutical research, viz. Chemistry, Materia Medica, Zoology, Botany, Mineralogy, &c.;" and, judging from the numbers which we have seen, we should say that the objects proposed by it are ably and zealously pursued. Although we find stated among the reasons for changing the name of this journal, that the number of subscribers were hardly sufficient to meet the necessary expenses, we must say that the very existence of such a journal, and its existence for a period of seven years, is not a little creditable to the zeal and intelligence of our American brethren. We may add, that we think it *not* creditable to the profession in our own country that there is no journal among us devoted to the same objects. Germany, France, Italy have all journals of the kind, some of them more than one; and we are unwilling to think so badly of the members of the profession in Great Britain as to believe that such a journal would not succeed among ourselves. We are willing to take the responsibility of recommending such a publication; and we hope some spirited members of our profession, learned in chemistry, materia medica, and pharmacy, will yet, as editors of such a journal, thank us for our suggestion.

The *American Journal of Pharmacy* consists of three departments,—original communications, selected articles, and miscellanies. In the first we observe several valuable articles on Botany and Pharmacy, and many things both useful and interesting to the practitioner and pharmaceutist. In the numbers before us, we have been struck by the numerous notices respecting the adulteration of medicines; and no better argument in favour of publications of this kind can be adduced, than the very fact of such adulterations being denounced, and the guilty agents exposed, in them. Certain it is that the adulteration of medicines has, in our own country, reached a most alarming height, and calls not only for the interference of the press, but for the strong arm of the law.

COLONIAL JOURNALS.

Under this head we shall include (whatever be the language in which they are composed,) the Medical Journals belonging to the colonies of all European countries; the whole, we believe, not forming a very long list. In a subsequent number we shall notice the only two English colonial journals with which we are acquainted, *The India Journal of Medical Science*, published in Calcutta, and *The Jamaica Physical Journal*, published in Kingston. On the present occasion we shall notice a journal of a *foreign* colony, (we believe we ought now to term it an independent state;) and some of our readers will probably be surprised to find any journal in a colony of which the mother-country (Portugal) is so low in general estimation for its medical institutions.

1.—*Revista Medica Fluminense, publicada pela Sociedade de Medicina do Rio de Janeiro.*

*The Rio Medical Review, published by the Medical Society of Rio Janeiro.—Rio de Janeiro,* 1835.

This journal appeared only last year, and the fourth number, now before us, is for July. It is in 8vo., printed in a very fair type, and on pretty good paper, greatly exceeding in this respect the Jamaica journal: it consists of three sheets, or forty-eight pages, appears monthly, and each number costs 600 *reis*, (about 3*s*.)

The journal, besides giving the minutes of the proceedings of the Society, contains original papers written by its members, and selections from European journals. The original papers are more numerous, and, we must add, more valuable, than we expected to find them. Among other articles, the July number contains a Case of Perforation of one of the Sigmoid Valves, with Observations; a Case of Pustula Maligna; a memoir on the Medicinal Properties of Tobacco; a notice of the Domestic and Medicinal Plants used in the Province of Pernambuco, &c. Among the rest, we observe a flaming Discourse of Senhor Dr. Soares de Meirelles, on the bad effects produced on the health of the living by "the passing bell" for the dead! According to the "Discurso" of the learned Senhor, these effects are really terrible. He conjures his auditors to set at nought all that ignorance and fanaticism may urge; and, like bold men and sage doctors, never cease until they have succeeded in putting an end to this barbarous immolation of the living to the dead! "Por tanto, senhores, ainda que a ignorancia e o fanatismo bradem contra vos, concorrei com vossas luzes e esforços à fim de que os vivos cessem de ser victimas dos mortos!" Verily, the bells of Rio de Janeiro must be of most terrific toll, or the fellow-townsmen of Senhor Soares marvellously faint-hearted, if it is indeed true, as he tells, (and he says he speaks "without hyperbole,") that the custom he denounces has sent thousands to their graves: "o qual (sem hyperbole) tem levado à tumba muitos milhares de homens!"

